# Neglected Tropical Diseases of Oceania: Review of Their Prevalence, Distribution, and Opportunities for Control

**DOI:** 10.1371/journal.pntd.0001755

**Published:** 2013-01-31

**Authors:** Kevin Kline, James S. McCarthy, Mark Pearson, Alex Loukas, Peter J. Hotez

**Affiliations:** 1 Departments of Pediatrics and Molecular Virology & Microbiology, and National School of Tropical Medicine, Baylor College of Medicine, Houston, Texas, United States of America; 2 Sabin Vaccine Institute and Texas Children's Hospital-Baylor College of Medicine Center for Vaccine Development, Houston, Texas, United States of America; 3 Clinical Tropical Medicine Laboratory, Queensland Institute of Medical Research, University of Queensland, Brisbane, Australia; 4 Centre for Biodiscovery and Molecular Development of Therapeutics, James Cook University, Cairns, Queensland, Australia; London School of Hygiene & Tropical Medicine, United Kingdom

## Abstract

Among Oceania's population of 35 million people, the greatest number living in poverty currently live in Papua New Guinea (PNG), Fiji, Vanuatu, and the Solomon Islands. These impoverished populations are at high risk for selected NTDs, including *Necator americanus* hookworm infection, strongyloidiasis, lymphatic filariasis (LF), balantidiasis, yaws, trachoma, leprosy, and scabies, in addition to outbreaks of dengue and other arboviral infections including Japanese encephalitis virus infection. PNG stands out for having the largest number of cases and highest prevalence for most of these NTDs. However, Australia's Aboriginal population also suffers from a range of significant NTDs. Through the Pacific Programme to Eliminate Lymphatic Filariasis, enormous strides have been made in eliminating LF in Oceania through programs of mass drug administration (MDA), although LF remains widespread in PNG. There are opportunities to scale up MDA for PNG's major NTDs, which could be accomplished through an integrated package that combines albendazole, ivermectin, diethylcarbamazine, and azithromycin, in a program of national control. Australia's Aboriginal population may benefit from appropriately integrated MDA into primary health care systems. Several emerging viral NTDs remain important threats to the region.

## Introduction

The neglected tropical diseases (NTDs) represent the most common infections of the world's poorest people, a group sometimes known as “the bottom billion” [1], [2]. These tropical infections trap people in poverty through their adverse effects on worker productivity, pregnancy outcomes, and child cognition and development [1], [2]. Recently, the World Health Organization (WHO) developed a list of 17 NTDs [3], with an expanded list of these conditions on the website of *PLOS Neglected Tropical Diseases* [4]. Since 2008, efforts have been made to review and describe the differences in the etiologies, prevalence, and disease burden of the major NTDs according to their regional distribution [5]–[12]. In this respect, the prevalence and distribution of the NTDs in the Americas [5]–[7], Europe [8], sub-Saharan Africa [9], China and East Asia [10], India and South Asia [11], Central Asia [12], and the Middle East and North Africa [13] have been previously reviewed. Here we summarize current knowledge on the prevalence and distribution of the NTDs in the region known as Oceania, which includes Australia, New Zealand, Melanesia, and the Polynesian and Micronesian islands of the Pacific. This review was conducted using the online database PubMed from 1997 to 2012 with the Medical Subject Headings, the specific diseases listed in the WHO's first report on NTDs, and the list from *PLOS Neglected Tropical Diseases* [3], [4], as well as the geographic regions and countries of Oceania. Reference lists of identified articles and reviews were also manually searched, as were databases from the WHO (http://www.who.int), including the *Weekly Epidemiological Record*.

## Poverty in Oceania

**Figure 1:**
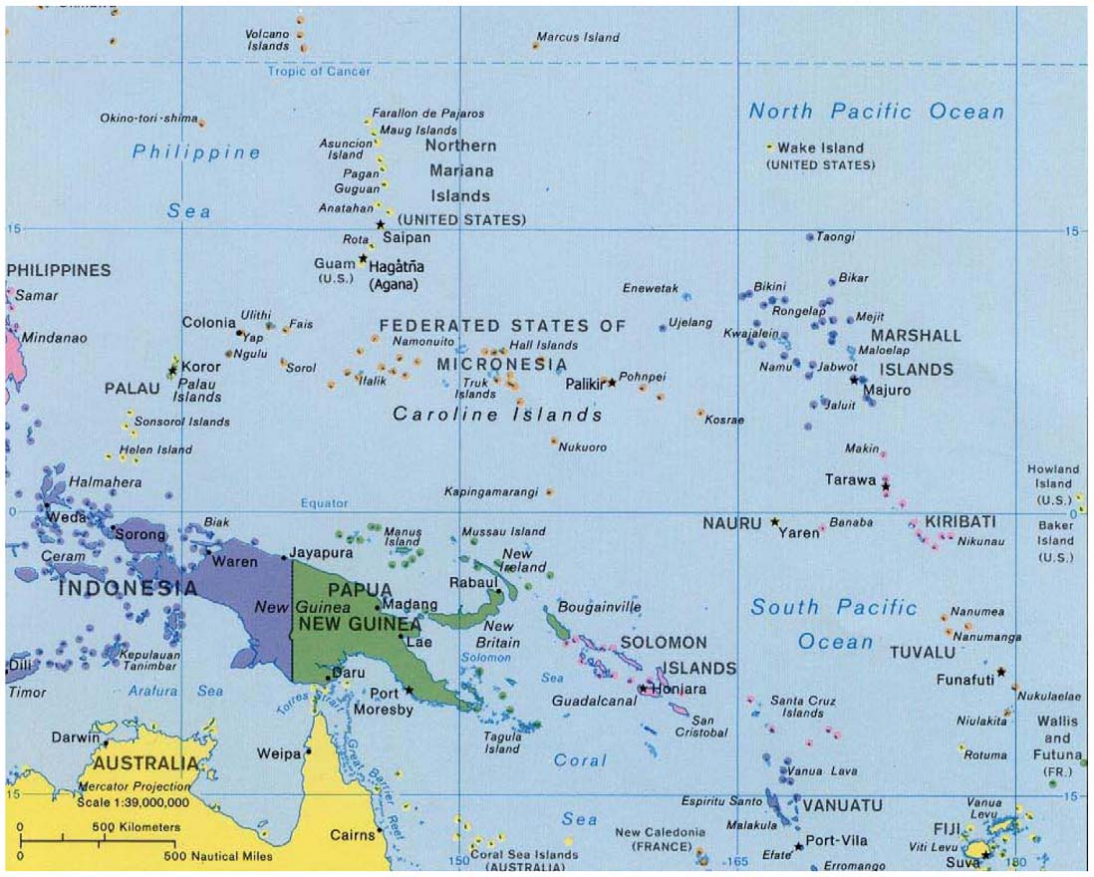
Map of West Pacific Islands. http://www.worldofmaps.net/en/oceania/map-pacific/map-west-pacific-islands.htm, accessed May 20, 2012.

Approximately 35 million people live in Oceania, a region of tropical and sub-tropical islands in the South Pacific Ocean (Figure 1). Almost two-thirds of the population (22.3 million) lives on the continent of Australia, followed in descending order by Papua New Guinea (6.8 million), New Zealand (4.4 million), Fiji (0.9 million), and the Solomon Islands (0.5 million) (Table 1). In all, of the dozens of island nations that comprise Oceania, more than 99% live in eight nations including those listed above, together with French Polynesia, New Caledonia, and Vanuatu. Despite their proximity to one another, the nations of Oceania represent a diverse array of economies. Australia and New Zealand each rank near the top of the United Nations human development indices (HDIs, 2nd and 5th, respectively) [14], whereas more than one-third of the population of Papua New Guinea (PNG) live below the World Bank poverty figure of US$1.25 per day [15]. PNG has an HDI of 153, placing it near the bottom of the global HDIs and is one of only four non-sub-Saharan countries (the others being Afghanistan, Haiti, and Yemen) with HDIs below 150 [14]. Fiji, Vanuatu, and the Solomon Islands also have HDIs of 100 or lower [14]. However, extremely impoverished indigenous groups are also found within the two wealthiest countries in the region, Australia and New Zealand. For instance, Aboriginal Australians, numbering nearly half a million, earn average incomes amounting to 62% of non-indigenous residents [16]. Within Australia, indigenous Australians reside in greatest numbers in New South Wales and Queensland, although the Northern Territory has the highest percentage of Aboriginal people [17].

**Table 1:** The Countries and Population of Oceania and the Percentage Living in Poverty.

Nation	Total Population	Percentage of Population Living below US$1.25 per Day	Percentage of Incidence of Poverty^a^ or Population below the Threshold for Relative Poverty^b^	Percentage of Population below 50% Median Income
Australia	22.3 million	N/A	N/A	N/A
Papua New Guinea	6.8 million	37.5 [15]	N/A	N/A
New Zealand	4.4 million	N/A	N/A	11 [123]
Fiji	860,000	31.0 [15]	35 [124]	N/A
Solomon Islands	538,000	N/A	22.7 [125]	N/A
French Polynesia	270,764	N/A	27.6 [126]	N/A
New Caledonia	254,000	N/A	N/A	17 [127]
Vanuatu	239,000	9.2 [128]	N/A	N/A

a Fiji, Solomon Islands.

b French Polynesia.

Throughout the world's low- and middle-income countries, and even among wealthy countries, the NTDs disproportionately affect people living in poverty, but especially those in extreme poverty [5]–[13]. Here we provide an overview of the major NTDs affecting people living in poverty in Oceania, with an emphasis on the eight nations with populations that exceed 200,000 and comprise more than 99% of the number of people living in this region.

## Helminth Infections

Helminth infections may represent the most prevalent NTDs in Oceania, led by hookworm and lymphatic filariasis (LF; Tables 2 and 3), although significant numbers of cases of ascariasis, trichuriasis, strongyloidiasis, and hymenolepiasis are also present.

**Table 2:** Major Helminth Infections in Oceania.

Disease^a^	Estimated Number of Cases in Oceania	Percentage of Global Disease Burden	Refs
Hookworm infection	5.5 million	1%	[18], [129]
Lymphatic Filariasis	2.7 million	2%	[34], [130]
Trichuriasis	1.2 million	<1%	[18], [129]
Ascariasis	1.2 million	<1%	[18], [129]

a Numbers for the hookworm infection, trichuriasis, and ascariasis were derived by multiplying the current population of each nation as reported in Table 1 by the percentage of people infected as reported in reference [18].

**Table 3:** Geographic Distribution of the Major Helminthic NTDs in Oceania.

Disease^a^	Country of Largest Prevalence (# of Cases)	Country of Second Largest Prevalence (# of Cases)	Country of Third Largest Prevalence (# of Cases)	Country of Fourth Largest Prevalence (# of Cases)	Refs
Hookworm	Papua New Guinea (4.9 million)	Fiji (318,000)	Solomon Islands (192,000)	Vanuatu (88,000)	[18]
Trichuriasis	Fiji (541,000)	Solomon Islands (338,000)	Papua New Guinea (204,000)	Vanuatu (150,000)	[18]
Ascariasis	Papua New Guinea (748,000)	Fiji (215,000)	Solomon Islands (135,000)	Vanuatu (59,000)	[18]
Lymphatic filariasis	Papua New Guinea (2.7 million)				[34]

a Numbers for the hookworm infection, trichuriasis, and ascariasis were derived by multiplying the current population of each nation as reported in Table 1 by the percentage of people infected as reported in reference [18].

### Soil-Transmitted Helminth Infections

Hookworm infection is possibly the most prevalent NTD in Oceania, with an estimated 5.5 million cases, comprising roughly 1% of the world's cases of hookworm infection [18]. Most of Oceania's hookworm cases are concentrated in PNG, where according to some estimates three-quarters of the population is infected, followed by Fiji, the Solomon Islands, and Vanuatu [18]. *Necator americanus* is the predominant hookworm species in PNG, comprising 100% of the hookworms in some areas [19], [20] (Supplemental Table 1 in Text S1) It is not known whether *Ancylostoma duodenale* is also present in PNG, but during the Australian Hookworm Campaign of 1919–1924, PNG was also studied, and *N. americanus* was found to be virtually the only hookworm detected [21]. In contrast, both *N. americanus* and *A. duodenale* may have been present historically in Australia among both white and Aboriginal communities. Today, hookworm is found almost exclusively among Aboriginal Australians in Western Australia and the Northern Territory, where *A. duodenale* is believed to be the sole species [21]. During the 1990s, isolated Aboriginal populations in northwest Australia exhibited hookworm prevalence rates that exceeded 75% (with high rates of hookworm anemia) [22], but no recent published data are available. However, it is likely that mass drug administration (MDA), although provided inconsistently, has reduced the hookworm prevalence among selected communities [23]. A unique eosinophilic enteritis syndrome caused by the dog hookworm, *Ancylostoma caninum*, has also been reported from north Queensland, and elsewhere in Australia, although it is not considered a significant public health problem [24].

Among the other soil-transmitted helminth nematode infections, ascariasis and trichuriasis are much less common in Oceania. However, trichuriasis appears to be a common geohelminth in Fiji, accounting for almost one-half of the number of cases in Oceania, while large numbers of cases also appear in the Solomon Islands and Vanuatu [18]. PNG accounts for much of the ascariasis in Oceania [25], followed by Fiji, the Solomon Islands, and Vanuatu [18]. Strongyloidiasis is an important soil-transmitted helminth infection in Oceania, although no overall prevalence data are available. Among Aboriginal Australian populations, the high rates of *Strongyloides stercoralis* infection may partly reflect a high incidence of human T-cell lymphotropic virus type 1 (HTLV-1) infections, which predisposes to this parasite. Prevalence rates of *S. stercoralis* infection as high as 60% have been reported [26]. A high mortality from *S. stercoralis* and HTLV-1 co-infections can result because of *Strongyloides* hyperinfection [27]. In a study of *Strongyloides* hyperinfection in central Australia among Australian Aboriginals, 77% were found to be HTLV-1 positive [28]. In PNG, *S. stercoralis* infection also occurs [26], as well as a unique form of strongyloidiasis caused by *S. fuelleborni kellyi* [25]. This form of the infection can be vertically transmitted, and has been associated with swollen belly syndrome in the Gulf and Madang provinces [25]. In one study, 27% of children tested positive for *Strongyloides*, with 81% of these children under the age of one [25]. Strongyloides-infected populations in both Australia and PNG may therefore benefit from MDA programs using ivermectin [26]. Outside of PNG, *S. stercoralis* infection has recently been described in medical volunteers in the Solomon Islands [29]. Sporadic cases of human *Trichostrongylus* infections have also been reported [30]–[32].

*Hymenolepis nana* infection has been reported as a common soil-transmitted cestodiasis among Aboriginal communities in Australia and PNG [23], [33], although the overall prevalence is not known. In Australia's Northern Territory, MDA with albendazole was found to be ineffective at reducing the prevalence of this infection [23].

### Lymphatic Filariasis (LF)

Next to hookworm and possibly strongyloidiasis, LF is likely to be the most prevalent helminthic NTD in Oceania. Although LF is found throughout Oceania, PNG is the only nation with a published national prevalence estimate [34]. In 1997 it was estimated that approximately 2.7 million people were infected [34], accounting for more than 2% of the global disease burden. Since then, MDA has been implemented with diethylcarbamazine citrate (DEC) plus albendazole [35]. On Lihir Island, MDA in conjunction with vector control led to a 75% reduction in the seroprevalence of microfilarial antigenemia [36], [37] and evidence of reduced transmission [38]. Despite these measures, the WHO reported in 2010 that, of the 5.6 million people in PNG who would benefit from empirical chemotherapy for LF, only 6.3% had been covered nationally [39].

Throughout most of Oceania, LF elimination efforts through MDA are underway through the auspices of the Pacific Programme to Eliminate Lymphatic Filariasis (PacELF) [40]. MDA has now ceased in Vanuatu because the prevalence has dropped below the 1% threshold. Ongoing post-MDA surveillance will be required to certify elimination efforts in this country [40]. On Ouvea Island, New Caledonia, a survey indicated that approximately one-third of the population was antigenemic prior to launching their PacELF program [41]. As of 2010, mapping of LF is complete in New Caledonia, but no MDA program has been implemented [39]. Fiji and French Polynesia currently have active MDA programs underway with 100% coverage [36]. On Fiji's Kadavi Island, DEC has reduced the percent testing positive for microfilaremia by 90% [42]. In PNG, PacELF has not yet been implemented at a national level. The remaining countries—Australia, New Zealand, and Solomon Islands—are considered non-endemic [36]. Of interest, the Solomon Islands achieved elimination largely through a program of vector control rather than MDA [43].

### Zoonotic Helminth Infections

Two emerging helminth zoonoses, cystic echinococcosis (hydatid disease) and cysticercosis, are of concern. While several eradication programs have been implemented on the mainland of Australia, *Echinococcus granulosus* remains widespread among sheep and macropods [44], [45]. In Tasmania, following a successful eradication program, only one echinococcosis case of mainland Australian origin has been reported since 1974 [46]. Although echinococosis has been declared eliminated in New Zealand, reports of hydatid disease presentations have occurred in Auckland, likely through importation [46], [47]. Cysticercosis is not endemic in Australia, but it has occurred in recent immigrants and Australians traveling to endemic regions [48], [49]. In PNG, indigenous and West Papuan refugees living along the border were found to have asymptomatic *Taenia solium* infections, but more comprehensive studies are needed to recognize the true prevalence of infection [50]. *Trichinella psuedospiralis* infections have been isolated from humans in Tasmania [51].

## Protozoan Infections

The major intestinal protozoan infections in Oceania are amebiasis, balantidiasis, cryptosporidiosis, and giardiasis (Supplemental Table 5). In Australia, these infections can disproportionately affect Aboriginal populations. However, no overall prevalence estimates are available. Among urban Australians, an analysis of seroprevalence of men who have sex with men (MSM) indicated that HIV+ MSM have a higher seroprevalence of *Entamoeba* sp. infection than HIV− MSM [52], [53]. It is uncertain whether this represents infection with the pathogenic species *Entamoeba histolytica* or the saprophyte *Entamoeba dispar*. In New Caledonia, *E. histolytica* was found among hospitalized patients with hepatic abscesses [54]. Epidemic foci of *Balantidium coli* infection have been reported from swine-producing areas of PNG, and an outbreak of balantidiasis was described after a typhoon on Truk resulted in contamination of ground and surface water with pig feces [55]. Giardiasis is also common among Aboriginal Australians and presumably other populations in Oceania [22]. Albendazole used for deworming these populations may also have had some activity against *Giardia* [56], [57]. Among non-Aboriginal communities, exposure to this pathogen has been identified in waterborne outbreaks, and is believed to be common among children in daycare settings [58]–[60]. In New Zealand, *Giardia* parasites were found in some people presenting with acute gastrointestinal illness [61]. Chagas disease is not endemic in any of the nations in the Oceania region. However, immigration of over 80,000 immigrants from Latin America to Australia in 2006 is likely to have resulted in the importation of 1,000 or more cases [62]. When combining the permanent arrivals from Argentina, Brazil, Chile, El Salvador, and Uruguay, as well as the resident population of Latin American immigrants from Columbia and Peru, the potential number of infected individuals in this group has been estimated to be 16 per 1,000 in Australia [63].

## Bacterial and Fungal NTDs

The major bacterial NTDs include the treponematoses, yaws, and congenital syphilis; intracellular bacterial infections including active trachoma, leprosy, Buruli ulcer, bartenellosis, bovine tuberculosis (TB), and brucellosis; and leptospirosis and cholera (Supplemental Tables 2–4, 6). Mycetoma is a fungal NTD in the region.

### Treponematoses

Yaws has not been eliminated from Oceania despite its susceptibility to MDA with azithromycin, or azithromycin used together with DEC in programs of PacELF [64], [65] (Supplemental Table 6). While great strides have been made in eliminating yaws, this chronic infection remains endemic in PNG, the Solomon Islands, and Vanuatu, although advanced cases are rare [66]. Infections occur among schoolchildren in PNG, including a high proportion of cases in the secondary stage (46%) [67], while an unspecified number of cases have been serologically detected in the Solomon Islands and elsewhere [68], [69]. Studies on school children from the island of Tanna in Vanuatu have recently confirmed a resurgence of yaws [70]. As part of WHO's elimination program for congenital syphilis, PNG, the Solomon Islands, and Fiji have reported surveillance data on this infection, with PNG exhibiting the highest prevalence [71]. A prior WHO report combined seven studies of maternal syphilis seroprevalence from 1997 to 2003 for Vanuatu. Studies of syphilis among pregnant women were also performed in New Caledonia with seroprevalence between 7% and 12.4% [72].

### Active Trachoma, Leprosy, and Other Intracellular Bacterial NTDs

Trachoma infections occur in Australia, PNG, Fiji, Vanuatu, and the Solomon Islands (Supplemental Table 2). The most recent released data from the WHO in 2003 indicated the greatest number of active trachoma cases were in PNG (16,289), followed by Australia (8,800), Fiji (1,865), and the Solomon Islands (1,403) [73]. Additional data indicated that the prevalence of active trachoma in Fiji, the Solomon Islands, and Vanuatu are similar (22%–23%) [74]. Aboriginal Australians living in remote communities also suffer from high prevalence of trachoma [75]. The SAFE (surgery, antibiotics, facial cleanliness, and environmental control) program has been implemented among Aboriginal Australians communities, with some reductions in overall prevalence [76].

The prevalence of leprosy is again highest in PNG, with 281 reported new cases in 2010 in addition to an estimated 580 existing cases [77]. Among Aboriginal Australians in the early 1950s, the incidence of diagnosis of leprosy was 270 per 100,000, but had fallen to 4/100,000 in the Northern Territory by 1997 [78]. This decrease was attributed to widespread use of the BCG vaccine in Aboriginal populations since 1958 [78]. Buruli ulcer, another mycobacterial infection, is endemic in some specific locations in Southeastern Australia and Queensland, with focal outbreaks being reported [79], [80]. Bartonellosis caused by *Bartonella henselae* has been detected in blood donors from Australia and New Zealand, as well as in children with hepatic abscesses in New Caledonia [81]–[84]. Bovine TB is present New Zealand, but it represents a small proportion of the overall TB incidence in Oceania [85], [86]. There were also cases of bovine TB originating in PNG that were detected in Australia [86]. Brucellosis is reportable in Australia, with 32 cases documented in 2009 [87].

### Leptospirosis

Leptospirosis is endemic in Oceania, with both sporadic cases and outbreaks being reported. In Australia, there has been a significant increase in the incidence of leptospirosis over the past decade, with the heaviest occupational burden among banana farmers and dairy workers [88]. In 2009, Australia reported 149 cases of leptospirosis nationally, with over 75% of the cases occurring in Queensland [87]. In New Zealand, 81 cases of leptospirosis were reported in 2010, with an elevated incidence in Ruapehu, the West Coast District, and Hawke's Bay [89], [90]. In New Caledonia, an outbreak of leptospirosis occurred during heavy rainfalls and flooding attributed to La Nina [91], while American Samoa was found to have a high prevalence of the disease [92]. Previously in Fiji, Vanuatu, and French Polynesia, *Leptospira icterohemorrahgiae* was identified as the dominant species [93], [94].

### Cholera

Cholera outbreaks have been noted in several nations of Oceania. In 2009, an outbreak occurred in PNG, eventually reaching 8,997 cases by the end of 2010, with the highest incidence in the Madang Province [95], [96]. In addition, an outbreak was reported from a resort in Fiji [97]. In Australia, four cases, all imported were reported in 2009.

## Arboviral Infections

The major arboviral infections in Oceania are the flavivirus infections caused by dengue, Japanese encephalitis (JE), and Murray Valley encephalitis (MVE), as well as mosquito-transmitted alphavirus infections, Ross River virus (RRV) and Barmah Forest virus (BFV).

Overall, the incidence of dengue is underreported in Oceania. In Australia, dengue infection and associated mortality were first identified in Charters Towers in northern Queensland in 1897 [98]. There have been reports of dengue in Fiji, French Polynesia, New Caledonia, the Solomon Islands, and Vanuatu prior to 1950 [99]. In 2010, the Western Pacific Region of the WHO (WPRO) reported national incidence data for dengue in Australia, Fiji, French Polynesia, New Caledonia, New Zealand, and Vanuatu (Supplemental Table 4) [100]. French Polynesia, New Caledonia, Vanuatu, and Australia accounted for more than 90% of the reported cases within the Western Pacific subregion [100]. In Australia, North Queensland reports the greatest number of cases [100], which included an outbreak during the 2008–2009 wet season [101]. Dengue is not endemic in New Zealand, and in 2010 all 51 reported cases in New Zealand were of foreign origin, with Vanuatu accounting for 12% of those cases [87], [100]. In addition to WPRO surveillance, a seroprevalence study indicated that dengue has also emerged in the Solomon Islands [102]. The overall epidemiology of dengue is perhaps least understood in PNG, although evidence for the infection has been found in adults and children with febrile illness [103].

Within Oceania, JE and MVE are found primarily in Australia and PNG. JE emerged during the 1990s in PNG and in the Torres Strait of Australia [104]. The reports of JE in the Torres Straight islands may have resulted from movement of migratory birds or wind-blown mosquitoes from PNG [105]. The JE isolates from PNG and Torres Strait share >99% sequence identity [106]. In Australia, MVE is endemic in north and southeastern Australia, with four cases reported 2009 [87], [107], while in PNG MVE was identified in mosquito isolates, but no human seroprevalence data are available [106].

In Australia, RRV infection has been reported periodically, but outbreaks have become more intense and frequent [108]. In 2009, 4,786 cases were reported in Australia, with nearly half in Queensland [87]. In PNG, no national prevalence data have been compiled, but in the Southern Highlands Province antibody prevalence for RRV was 59% [109]. After an apparent disappearance in the years following a 1979–1980 outbreak, RRV has reemerged in Fiji [110]. BFV is unique to Australia, and is distributed throughout the continent with the highest incidence in Queensland [111]. In 2008, the national incidence of BFV was found to have increased 34% over the mean rate of the previous 5 years [111]. Within Australia, RRV and BFV account for most of the reported arbovirus disease notifications [101].

## Ectoparasitic Infections

Scabies is a major endemic ectoparasitic infection among Aboriginal Australians and in other Oceanic nations. In Australia, pre-treatment prevalence levels exceeded 30% among indigenous children in some communities [112], [113], with high rates of secondary infections of streptococcal pyroderma [112]–[114]. MDA treatment with ivermectin in a village in PNG was found to reduce scabies prevalence by two-thirds to 26% [115]. In the Solomon Islands, scabies prevalence among children was reduced with ivermectin treatment to 0.7% [116]. In two studies undertaken in Fiji, the burden of scabies in schoolchildren was between 18.5% and 32% [117], [118]. A study in Vanuatu demonstrated increased efficacy of ivermectin over benzyl benzoate in treating childhood scabies [119]. Myiasis has also been reported in New Zealand, acquired both in the country and internationally [120].

## Discussion

Several important NTD trends have emerged in the Oceania region.

1. *MDA.* Proof of concept for achieving success in MDA in Oceania has been obtained through the PacELF with high levels of LF treatment coverage in Fiji, French Polynesia, and New Caledonia, and the possible elimination of LF in Vanuatu [40]. The Solomon Islands has previously eliminated LF through vector control [43]. As such successes have not yet extended to PNG, it remains the most endemic in Oceania [36], [40]. A concerted effort in PNG for LF elimination in the coming years would be consistent with efforts by the Global Programme to Eliminate LF's to eliminate this disease as a public health problem globally by 2020 [40], [121]; there is an urgency to ensure that the government of PNG has the adequate human capital and technical support as well as sufficient funding to expand MDA and elimination efforts. Currently, there are no comprehensive post-MDA surveillance programs in place for any LF-endemic country in the region. The development and implementation of surveillance plans for all countries will be important to monitor areas of persistent or re-emerging LF [40]. Additional targets for MDA in Oceania could include soil-transmitted helminth infections, which could be linked to LF elimination efforts through the addition of albendazole to DEC, as well yaws elimination through the addition of azithromycin to DEC [64], [65]. The recent finding by Mitja et al. that single-dose azithromycin is as effective as benzathine benzyl penicillin for the treatment of yaws in PNG is an important breakthrough on that front [64]. There are opportunities to scale up these options, especially in PNG [64].
2. *PNG.* More than any other nation in Oceania, PNG stands out with respect to having the largest number of cases and high prevalence of several key NTDs, including *N. americanus* hookworm infection, strongyloidiasis, hymenolepiasis, LF, balantidiasis, yaws, trachoma, leprosy, and possibly scabies and dengue and other arboviral infections, as well as outbreaks of cholera [18], [25], [33], [34], [55], [64], [73], [77], [103], [115]. PNG could benefit enormously from a national control program of integrated MDA that simultaneously targets many of these NTDs using the drugs albendazole, ivermectin, DEC, and azithromycin. Therefore, there are opportunities to collect the safety data needed to determine the suitability of combining these medicines in an integrated MDA package, together with financial support for implementation linked to operational research.
3. *Aboriginal populations in Australia*. Australia's Aboriginal population also suffers from disproportionately high rates of NTDs, including strongyloidiasis, leprosy, and scabies, and possibly hookworm infection [26], [73], [112], [113]. There may be opportunities for integrating MDA packages as outlined above for PNG into programs of primary care for Australia's Aboriginal populations.
4. *Arboviral infections*. The impact of arboviral infections, especially emerging dengue and RRV in PNG, is still not well understood [106]. Dengue, JE, and RRV could emerge as important NTD pathogens in Oceania in the coming decade. Malaria is endemic in regions of PNG, the Solomon Islands, and Vanuatu. In the Solomon Islands, malaria vector control was responsible for a substantial reduction of LF [122]. Current malaria programs implemented by the Pacific Malaria Initiative Support Centre (PacMISC) in Vanuatu and the Solomon Islands as well as the Global Fund in PNG are promising tools for reducing the burden of several mosquito-borne diseases, particularly in PNG, where *Anopheles* transmit both malaria and LF. Integration of monitoring and evaluation M&E in these programs and in non-endemic nations will provide assistance in controlling the rising threat of arboviral pathogens in the future.

In summary, tremendous strides have been made in controlling and eliminating selected NTDs in Oceania, but mostly through PacELF. There is an urgent need to extend these successes to all of the major NTDs throughout the region but especially in PNG and among Aboriginal Australians.

**Learning Points**

- The efficacy of the Pacific Programme to Eliminate Lymphatic Filariasis (PacELF) has been demonstrated in Oceania, but enhanced efforts are still required in Papua New Guinea and New Caledonia.
- While proof of concept for NTD management in Oceania was shown by PacELF, novel MDA programs need to be crafted, especially in Papua New Guinea and Aboriginal Australia, in order to target specific NTDs indigenous to neglected populations in these regions, including hookworm, strongyloidiasis, and other soil-transmitted helminthiases, yaws, trachoma, and scabies.
- While the impact of emerging arboviral infections, including dengue, Japanese encephalitis, and Ross River virus infection, is still not well understood, these diseases could also emerge as important NTDs in the coming decade.

**Key Articles in the Field**

1. King SE, Mascie-Taylor CG (2004) Strongyloides fuelleborni kellyi and other intestinal helminths in children from Papua New Guinea: associations with nutritional status and socioeconomic factors. PNG Med J 47: 181–191.
2. Weil GJ, Kastens W, Susapu M, Laney SJ, et al (2008) The impact of repeated rounds of mass drug administration with diethylcarbamazine plus albendazole on Bancroftian filariasis in Papua New Guinea. PLoS Negl Trop Dis 2: e344. doi:10.1371/journal.pntd.0000344
3. Andrews RM, Kearns T, Connors C, Parker C, et al. (2009) A regional initiative to reduce skin infections amongst Aboriginal children living in remote communities of the Northern Territory, Australia. PLoS Negl Trop Dis 3: e554. doi:10.1371/journal.pntd.0000554
4. Van den Hurk AF, Craig SB, Tulsiania SM, Jansen CC (2010) Emerging tropical diseases in Australia. Part 4. Mosquitoborne diseases. Ann Trop Med Parasitol 104: 623–640.
5. Mitja O, Hays R, Ipai A, Penias M, Paru R, Fagaho D, et al. (2012) Single dose azithromycin versus benzathine benzylpenicillin for treatment of yaws in children in Papua New Guinea: an open-label, non-inferiority, randomised trial. Lancet 379: 342–347.

## Supporting Information

Text S1 Supporting file containing Supplemental Tables 1–7.
(DOC)
