# Supplementary material for: Neglected Tropical Diseases of Oceania: Review of Their Prevalence, Distribution, and Opportunities for Control
Source: PLoS Negl Trop Dis. 2013 Jan 31;7(1):e1755. doi: 10.1371/journal.pntd.0001755 (PMC3561157; doi:10.1371/journal.pntd.0001755)
Supplement: Text S1 — Supporting file containing Supplemental Tables 1–7. (DOC) [file pntd.0001755.s001.doc]

Supplemental References

Supplemental Table 1. Soil-transmitted helminth infections in Oceania

| Neglected infection | Country | Special populations studied | [Reference] |
| --- | --- | --- | --- |
| Hookworm | Aboriginal Australia | Children in Aboriginal Community in Northwest Australia | [22] |
|  |  | Aboriginal Community in Northwest Australia | [22] |
|  |  | Prevalence in Western Australia Community after large-scale chemotherapy | [23] |
|  |  | Prevalence in Kimberley, Northwest Australia | [56] |
|  |  | Coastal community in Western Australia | [57] |
|  | Australia | Cases of Ancylostoma caninum in Queensland | [24] |
|  | Papua New Guinea | Prevalence in Kabasob village | [20] |
|  | Fiji | Schoolchildren of Taveuni Island | [118] |
|  |  | Schoolchildren | [131] |
|  | Solomon Islands | Schoolchildren | [131] |
|  | Vanuatu | Schoolchildren | [131] |
|  |  |  |  |
| Trichuriasis | Aboriginal Australia | Prevalence in Kimberley, Northwest Australia | [56] |
|  |  | Adults and children in East Arnhem, Northern Territory | [114] |
|  | Fiji | Schoolchildren of Taveuni Island | [118, 131] |
|  | French Polynesia | Schoolchildren | [131] |
|  | Solomon Islands | Schoolchildren | [131] |
|  | Vanuatu | Schoolchildren | [131] |
|  |  |  |  |
| Ascariasis | Aboriginal Australia | Children 5-14 | [22] |
|  | New Caledonia | Retrospective study of childhood hepatic abscess hospitalizations | [54] |
|  | Fiji | Schoolchildren of Taveuni Island | [118] |
|  |  | Schoolchildren | [131] |
|  | Solomon Islands | Schoolchildren | [131] |
|  | Vanuatu | Schoolchildren | [131] |
|  |  |  |  |
| Strongyloidiasis | Aboriginal Australia | Prevalence in Kimberley, Northwest Australia | [56] |
|  |  | Prevalence of S. stercoralis in Indigenous Australians | [26] |
|  |  | HTLV-1 (+) percentage of presentation with hyperinfection | [28] |
|  | Papua New Guinea | Children near Kanabea | [25] |
|  |  | Prevalence of S. stercoralis | [26] |
|  | Solomon Islands | Medical volunteers | [29] |
|  |  |  |  |
| Lymphatic Filariasis | Papua New Guinea | Before MDA on Lihir Island | [38] |
|  |  |  |  |
|  |  | After MDA in rural villages in Madang | [35] |
|  |  | After Filariasis control program on Lihir Island | [37] |
|  |  | National prevalence rate | [34] |
|  |  | Population requiring preventative chemotherapy | [39] |
|  | Fiji | Adults after five annual single doses of diethylcarbamazine | [42] |
|  |  | Population requiring preventative chemotherapy | [39] |
|  | New Caledonia | Seropositive individuals on Ouvea Island | [41] |
|  |  | Population requiring preventative chemotherapy | [39] |
|  | Vanuatu | Following two years of MDA treatment | [133] |
|  | French Polynesia | Adults and children | [134] |
|  |  | Population requiring preventative chemotherapy | [39] |
|  |  |  |  |
| Hymenolepiasis | Australia | Fecal samples | [135] |
|  | Aboriginal Australia | Northern Australian community prevalence | [23] |
|  | Papua New Guinea | Human infection | [33] |
|  |  |  |  |
|  |  |  |  |

Supplemental Table 2. Geographic Distribution of Active Trachoma and Leprosy in Oceania

| Disease | Country of Highest Incidence (#of Cases) | Country of Second Highest Incidence (# of Cases) | Country of Third Highest Incidence  (# of Cases) | Country of Fourth Highest Incidence (# of Cases) | [Refs] |
| --- | --- | --- | --- | --- | --- |
| Trachoma | Papua New Guinea (16,289) | Australia (8800) | Fiji (1865) | Solomon Islands (1403) | [73] |
| Leprosy | Papua New Guinea (281) | Solomon Islands (14) | Australia (9) | New Caledonia (8) | [77] |

Supplemental Table 3. Other Bacterial and Fungal NTDs

| Neglected infection | Country | Special Populations Studied | [Reference] |
| --- | --- | --- | --- |
| Trachoma | Aboriginal Australia | Children in endemic village with trachomatous inflammation-follicular | [136] |
|  |  | Active trachoma in Aboriginal children 1-9 | [75] |
|  |  | Active trachoma prevalence post intervention | [76] |
|  | Australia | Annual cases | [73] |
|  | Papua New Guinea | Annual cases | [73] |
|  | Fiji | Average rate of active trachoma | [74] |
|  |  | Annual cases | [73] |
|  | Solomon Islands | Average rate of active trachoma | [73] |
|  |  | Annual cases | [73] |
|  | Vanuatu | Average rate of active trachoma | [74] |
|  |  | Annual Cases | [73] |
|  |  |  |  |
| Leprosy | Aboriginal Australia | Adults and children in Northern Territory | [78] |
|  | Australia | Number of new cases (2010) | [77] |
|  | New Zealand | Number of new cases (2010) | [77] |
|  | New Caledonia | Number of new cases (2010) | [77] |
|  | Papua New Guinea | Number of new cases (2010) | [77] |
|  | Fiji | Number of new cases (2010) | [77] |
|  | Solomon Islands | Number of new cases (2010) | [77] |
|  | Vanuatu | Number of new cases (2010) | [77] |
|  | French Polynesia | Number of new cases (2010) | [77] |
|  |  |  |  |
| Buruli Ulcer | Australia | Incidence in Victoria | [79] |
|  |  | Mean annual incidence, Port Lonsdale | [80] |
|  |  |  |  |
| Bartonella | Australia | Adults and children | [81] |
|  |  | Blood donors in New South Wales | [82] |
|  | New Zealand | Blood donors | [83] |
|  | New Caledonia | Children with hepatic abscesses | [84] |
|  |  |  |  |
| Bovine Tuberculosis | New Zealand | M. bovis infection incidence among cases of M. tuberculosis infection | [85] |
|  | Papua New Guinea | Proportion of Australian cases of M. tuberculosis of PNG origin | [86] |
|  |  |  |  |
| Brucellosis | Australia | Cases reported nationally | [87] |
|  |  |  |  |
| Leptospirosis | Australia | Incidence in Queensland | [137] |
|  |  | Cases reported nationally | [87] |
|  | New Zealand | Changing epidemiology of human leptospirosis | [90] |
|  |  | Slaughterhouse workers | [138] |
|  |  | Intensive care unit | [90] |
|  |  | Notifiable disease rate | [89] |
|  | Fiji | Leptospirosis suspect patients | [93] |
|  | New Caledonia | Outbreak | [91] |
|  |  | Average incidence 2001-2005 | [139] |
|  | Vanuatu | Hospital admissions | [140] |
|  |  | Laboratory confirmed cases | [93] |
|  | French Polynesia | Annual incidence | [94] |
|  |  |  |  |
| Cholera | Australia | Cases reported nationally | [87] |
|  | Papua New Guinea | Village attack rates | [95] |
|  |  | National outbreak | [96] |
|  | Fiji | Outbreak | [97] |
|  |  |  |  |
|  |  |  |  |

Supplemental Table 4. Arboviral NTDs

| Neglected infection | Country | Special population | [Reference] |
| --- | --- | --- | --- |
| Dengue | Australia | Number of cases for 2010 | [100] |
|  | New Zealand | Number of cases for 2010 | [100] |
|  | Papua New Guinea | Adults and children with acute febrile illness | [103] |
|  | Fiji | Number of cases for 2010 | [100] |
|  | New Caledonia | Number of cases for 2010 | [100] |
|  | Solomon Islands | IgG antibodies specific for dengue | [102] |
|  | French Polynesia | Number of cases for 2010 | [100] |
|  | Vanuatu | Number of cases for 2010 | [100] |
|  |  |  |  |
| Murray Valley Encephalitis | Australia | National incidence 2009 | [87] |
|  | Papua New Guinea | Isolates from mosquitoes | [107] |
|  |  |  |  |
| Ross River Virus | Australia | National incidence 2009 | [87] |
|  | Papua New Guinea | Antibody prevalence in Southern Highlands Province | [109] |
|  | Fiji | Serologically positive cases | [110] |
| Barmah Forest Virus | Australia | Annual notification rate for 2007/2008 | [111] |

Supplemental Table 5. Protozoan NTDs in Oceania

| Neglected infection | Country | Special populations studied | [Reference] |
| --- | --- | --- | --- |
| Amebiasis | Australia | High risk MSM | [52, 53] |
|  | New Caledonia | Retrospective study of childhood hepatic abscess hospitalizations | [54] |
|  |  |  |  |
| Balantidiasis | Papua New Guinea | Pig farmers | [33] |
|  |  |  |  |
| Giardiasis | Aboriginal Australia | Aboriginal Community in Northwest Australia | [56] |
|  |  | Coastal community in Western Australia | [57] |
|  | Australia | Waterborne outbreaks of GI disease | [60] |
|  |  | Highly credible GI events | [58] |
|  | New Zealand | Acute gastroenteritis patients | [61] |
|  |  | Average annual infection rate | [141] |
| Chagas Disease | Australia | Latin American immigrants | [63] |
|  |  |  |  |

Supplemental Table 6. Treponematoses in Oceania

| Neglected infection | Country | Special populations studied | [Reference] |
| --- | --- | --- | --- |
| Yaws | Papua New Guinea | Children with clinically suspected Yaws | [67] |
|  |  | Cases in periurban inhabitants of Port Moresby over 18 months | [142] |
|  | Solomon Islands | Blood donors | [69] |
|  | Vanuatu | School children with Yaws-like skin lesions | [70] |
|  | French Polynesia | Serological and clinical survey | [68] |
| Syphilis | Papua New Guinea | % of women attending antenatal care seropositive for syphilis | [71] |
|  |  |  |  |
|  | Fiji | % of women attending antenatal care seropositive for syphilis | [71] |
|  | New Caledonia | Pregnant women | [72] |
|  |  |  |  |
|  | Solomon Islands | % of women attending antenatal care seropositive for syphilis | [71] |
|  | Vanuatu | Antenatal women | [143] |
|  |  |  |  |

Supplemental Table 7. Zoonotic Helminth Infections

| Neglected infection | Country | Special population | [Reference] |
| --- | --- | --- | --- |
| Echinococcosis | Australia | Campers and livestock workers | [44] |
|  |  | Retrospective analysis of new cases in New South Wales and Australian Capital Territory, 1987-1992 | [45] |
|  | New Zealand | Hydatid disease presentation in patients requiring surgical treatment of cystic liver lesions | [47] |
|  |  |  |  |
| Cysticercosis | Australia | Neurocysticercosis of Tanzanian origin | [49] |
|  | Papua New Guinea | Residents near border of Indonesia with Taenia solium | [50] |
|  |  |  |  |
| Trichostrongyliasis | Australia | Retrospective analysis of fecal examinations in Queensland 1992-1995 | [30] |
|  |  | Case report in suburban goat keepers | [31] |
|  | New Zealand | Outbreak among British tourists after visiting sheep farm | [32] |

Supplemental Table 8. Ectoparasitic NTDs in Australia

| Neglected infection | Country | Special Populations Studied | [Reference] |
| --- | --- | --- | --- |
| Scabies | Australia | Hospital admissions with S. aureus | [144] |
|  | Aboriginal Australia | East Arnhem children | [114, 145] |
|  |  |  |  |
|  |  | Minjilang population after treatment with permethrin cream | [113] |
|  |  |  |  |
|  | Papua New Guinea | Adults and children above the age of 5 in village, five months after MDA treatment | [115] |
|  | Fiji | Schoolchildren of Taveuni Island | [118] |
|  |  | School children | [117] |
|  |  | Infants | [117] |
|  | Solomon Islands | Children after ivermectin treatment | [116] |
|  | Vanuatu | Children | [119] |
|  |  |  |  |
| Myiasis | New Zealand | Retrospective study | [120] |
